# The Fiscal Consequences for the Canadian Government of Efgartigimod in the Treatment of Generalized Myasthenia Gravis

**DOI:** 10.36469/001c.157709

**Published:** 2026-03-11

**Authors:** Ana Teresa Paquete, Mark P. Connolly, Cynthia Qi, Hans Katzberg, Syed Raza, Charles Kassardjian, Zaeem A. Siddiqi, Roger Kaprielian, Jason Locklin, Glenn A. Phillips, Nikos Kotsopoulos

**Affiliations:** 1 Global Market Access Solutions LLC, Mooresville, North Carolina, USA; 2 argenx US Inc, Boston, Massachusetts, USA; 3 University Health Network, University of Toronto, Toronto, Ontario, Canada; 4 argenx UK Ltd, Mylton Keynes, United Kingdom; 5 epartment of Neurology, Department of Medicine University of Toronto, Toronto, Ontario, Canada; 6 Division of Neurology University of Alberta, Edmonton, Alberta, Canada; 7 argenx, Toronto, Ontario, Canada

**Keywords:** generalized myasthenia gravis, public economics, fiscal analysis, economic evaluation

## Abstract

**Background:** Generalized myasthenia gravis (gMG) severely impacts activities of daily living. Productivity losses and the need for care can impact household finances and consequently government public accounts. This study adopts a governmental perspective framework to assess the fiscal consequences of treating gMG that is inadequately controlled by standard therapy, beyond healthcare costs. Savings in tax revenue loss and benefit payments are considered. **Objectives:** To value the fiscal consequences of treating adults with acetylcholine receptor–antibody positive (AChR-Ab+) gMG with efgartigimod vs current treatments. The lifetime impact on people living with gMG and their caregivers is considered from the perspective of Canada’s public accounts. **Methods:** A lifetime Markov cohort simulation following adults with gMG according to their Activities of Daily Living (MG-ADL) score was linked to labor and fiscal stages of both patients and care- givers. Based on the MyRealWorld MG study, MG-ADL scores defined the labor market characteristics of both individuals with gMG and their caregivers. National statistics data on sex- and age-specific labor outcomes were used to model patients with minimal symptoms. Benefit payments and tax revenue losses attributable to gMG were estimated and valued according to national official sources. Public healthcare costs were included. The difference between efgartigimod and current treatments was assessed by discounted lifetime incremental fiscal consequences. Sensitivity analyses were applied to the fiscal parameters. **Results:** Without active treatments, the lifetime fiscal burden associated with individuals with gMG and their caregivers was estimated at CAD1.24millioningovernmentexpenditures.ComparedwiththecurrentweightedbundleoftreatmentsinCanada,efgartigimodwasestimatedtosaveCAD458 754 per treated adult. Results were sensitive to the distribution of the bundle of treatments. **Discussion:** Beyond healthcare costs, gMG severely impacts productivity and governmental accounts. Decision-makers should be provided evidence of fiscal consequences when assessing healthcare technologies. The public sector in Canada was estimated to have a return of CAD1.58pereveryCAD1 spent on efgartigimod for people with gMG compared with the current bundle of treatments. **Conclusions:** Improving health outcomes and reducing the need for informal caregivers benefits those affected by gMG and governmental accounts.

## BACKGROUND

Generalized myasthenia gravis (gMG) is a chronic autoimmune neuromuscular disease that leads to muscle weakness and fatigue. The range of symptoms in people with gMG causes a myriad of productivity consequences including absenteeism, presenteeism, discontinuation of work, and early retirement due to poor health and disability.^1–5^ These economic effects can be pronounced, as the onset of gMG symptoms often occurs in prime working ages, especially in women.^6^ Among employed persons with gMG, indirect costs often represent more than one-third of the overall cost burden.^7^ Furthermore, productivity losses can extend to other family members, with moderate to severe household economic problems being reported.^5^ For example, more than 40% of individuals with gMG require home care, often provided by family members who may forego paid work.^2^

The economic effects of gMG are not isolated to persons with the condition and their immediate family members. The losses of individual workers due to poor health can influence other economic domains, including public-sector accounts.^8^ Previous assessments of the impact of poor health from the governmental perspective suggest that healthcare costs are often a minor component of poor health, and lost tax revenue and workless benefits represent the main illness-related costs for the public sector.^9^

Disease management and treatment efficacy in gMG can have a key role in a household’s burden and family members’ work status. Management of gMG is mostly focused on symptom control and minimizing treatment-related adverse events. The standard of care (SoC) treatment in Canada includes therapies like acetylcholinesterase inhibitors, corticosteroids, and nonsteroidal immunosuppressive therapies. Despite these treatments, disease symptoms and treatment-related morbidities persist in many gMG patients, impacting their quality of life. Maintenance therapy with immunoglobulins (chronic IG), and plasma exchange or plasmapheresis are considered in patients whom disease is inadequately controlled by standard therapy.^10^ Adults whose acetylcholine receptor–antibody positive (AChR-Ab+) gMG is inadequately controlled are more likely to experience persistent symptoms.^3^

One of the latest therapies introduced for treating AChR-Ab+ gMG is efgartigimod alfa, which has shown clinically meaningful and sustained improvements in the Myasthenia Gravis–Activities of Daily Living scale (MG-ADL) and in health-related quality-of-life measures when added to SoC medication in patients whose symptoms persisted despite a stable dose of SoC medication (ADAPT trial).^3,11^ The MG-ADL scale assesses symptoms and routine daily activities and has been widely accepted among clinicians and decision-makers for assessing the disease activity and control.^12–14^ Improvements in activities of daily living suggest that working-age individuals on this treatment will be more likely to remain in the workforce, decrease their caregiving needs, and reduce the disease burden.^15–17^ Consequently, a broader impact is expected on the public economy, making it relevant to assess overall fiscal consequences of the novel treatment for decision-makers in Canada.

To gauge the full range of fiscal consequences of different gMG interventions and how likely they are to impact the economic life cycle, we developed a government perspective fiscal analysis. This framework considers how different administration investment choices can influence public accounts based on changes in productivity and spending on social programs. The analysis helps to illustrate the cross-sectorial public economic impact of different investment choices. Assessing broader fiscal consequences is highly important in countries like Canada, in which healthcare and social benefits are mostly funded by federal and provincial governmental taxation.

The main aim of the current study was to assess the fiscal consequences of adults living with AChR-Ab+ gMG whose symptoms persist despite stable doses of SoC medication and of their informal caregivers (iCGs) to Canada’s public accounts. The impact of different treatment options used before efgartigimod approval were compared: SoC, chronic IG, and efgartigimod. To simulate the scenario in the absence of efgartigimod, we considered a weighted comparator consisting of 75% on chronic IG and 25% on SoC alone.^18^

## METHODS

### Model Overview

The current fiscal analysis was based on a previously presented lifetime Markov cohort simulation,^19^ reviewed by the Canadian Drugs Agency (CDA).^10^ In this model, gMG patients’ lifetime was modeled according to MG-ADL scale categories (<5; 5-7; 8-9; ≥10), MG crisis event, and death **(Supplementary Figure S1)**. Exacerbations and treatment-related adverse events were also modeled, as well as corticosteroid use, with an increased impact on mortality. Time in each MG-ADL health state was used to assess the effectiveness of different therapeutic strategies: SoC alone, chronic IG in addition to SoC, and efgartigimod in addition to SoC. The administration regimens and dosing intensity reported in Siddiqi et al were adopted for this analysis.^19^

**Figure 1.**
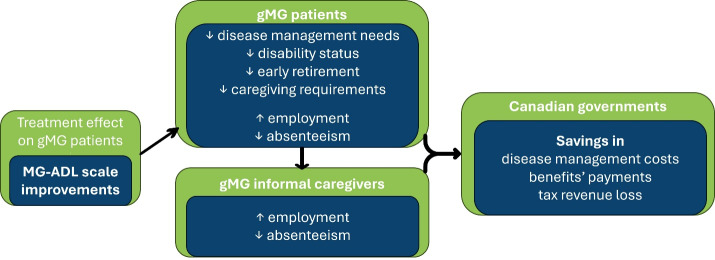
Fiscal Model Framework Abbreviations: gMG, generalized myasthenia gravis; MG-ADL, Myasthenia Gravis–Activities of Daily Living.

The fiscal model linked health states to the expected labor status of individuals with gMG and iCGs. The rationale behind the fiscal model was based on the premise that effective treatments have a positive impact on labor outcomes of people living with gMG, thus reducing their needs for home care. From the public sector perspective, productivity gains due to treatment prevent the loss of tax revenue for both federal and provincial governments. Additionally, the decreased need for sick leave benefits, disability pensions, early retirement pensions, and caregiving benefits reduces government expenditure. The fiscal model framework is illustrated in **Figure 1**. As the healthcare costs have previously been reported, the focus of the present analysis was on how different interventions influence social benefits programs and tax revenue losses.

Three pairs of cohorts, comprising individuals with gMG and iCGs, were modeled, according to the administered treatment: SoC alone, SoC + chronic IG and SoC + efgartigimod. An additional weighted cohort was created, considering that, without efgartigimod, 25% of the target population remained on SoC alone and 75% used SoC + chronic IG (weighted comparator).

The fiscal model started when patients initiated treatment with efgartigimod or chronic IG. For patients receiving the former treatment, the average age at treatment initiation was 47 years, according to the ADAPT study.^3^ Based on the differences registered in the MyRealWorld (MRW) MG study,^20^ iCG patients were 3 years older than patients receiving efgartigimod. Therefore, iCGs were considered to enter the model at 50 years of age. Subsequently, these cohorts were followed until the patients’ deaths.

The fiscal outputs generated were the expected lifetime fiscal consequences to the public sector in Canada by each cohort. Specifically, the fiscal consequences quantified were tax revenue from labor outcomes (absenteeism and workforce participation), government benefit payments (sickness benefits, disability benefits, retirement pensions and caregiving benefits), and healthcare costs (previously presented^10,19^) of both individuals with gMG and iCGs.

In addition to lifetime fiscal consequences (fiscal burden of disease) for each cohort, incremental consequences were also generated. It was expected that the most effective intervention for public investment would provide the greatest savings in terms of disease management costs, government benefit payments, and averted gMG-attributable tax revenue losses. Benefit-cost ratios were estimated, presenting the ratio of incremental fiscal consequences (to drug-related costs).

All fiscal outcomes were discounted at 3%, according to Canada’s cost-benefit analysis guide for regulatory proposals,^21^ whereas healthcare costs were discounted at 1.5%, based on CDA requirements on reimbursement reviews.^22^ An annual wage growth was applied to pro-ject future employment income, based on national historical data^23^ and social benefits paid by the government were assumed to increase at an annual growth rate equal to the Consumer Price Index.^24^

### Labor and Fiscal Stages Stratified by MG-ADL Scores

The labor and fiscal stages considered for people living with gMG are employed (on or off sick leave), disabled, and retired. Informal CGs were distributed according to the following fiscal stages: employed (full-time or reduced hours due to caring) and out of the workforce and consequently entitled to caregiving benefits. Health states were then linked to the labor and fiscal stages throughout patients’ lifetime horizon (**Supplementary Table S1)**.

The distribution of people living with gMG by labor and fiscal stages assumed that those in a controlled health state (MG-ADL <5) were considered similar to the demographically identical group in the Canadian general population. Therefore, this group was assumed to follow the sex- and age-specific probability of being employed, average wage, and average productivity levels of the Canadian population.^25–27^ Expected differences in scoring higher at the MG-ADL scale were based on the MRW MG study.^15^

The relative risks (RRs) of being employed or unable to work due to the disease for people living with gMG, based on the MRW MG study,^5,28^ are presented in **Table 1.** It was less likely that people who scored high on the MG-ADL symptom scale were employed. These RRs were applied to sex- and age-specific employment rates in the general population,^25^ assuming a maximum working age of 75 years. Productivity losses of employed adults with gMG were based on the frequency and time of sick leave, by MG-ADL scores.^15^

**Table 1.** People Living with gMG: Impact on Labor Market by MG-ADL Score

**MG-ADL Score**	**People Living with gMG^5,29^**		**People Living with gMG and Employed**	
	**RR of Being Employed**	**RR of Retiring Early or Unable to Work Due to gMG**	**% Taking Sick Leave^a^^17^**	**Average Time on Sick Leave (Days per Month)^15,29^**
<5	1	1	19	13.3
5-7	0.89	3.21	27	13.5
8-9	0.89	3.53	33	13.5
≥10	0.78	5.00	63	21.5

Abbreviations: gMG, generalized myasthenia gravis; MG-ADL, Myasthenia Gravis–Activities of Daily Living; MRW, MyRealWorld.

^a^Based on the odds ratio of taking sick leave by MG-ADL score (1.09).

^b^Due to the low numbers, categories MG-ADL 5-7 and 8-9 were jointly assessed.

Data from MRW MG Study were also used to identify the proportion of people living with gMG requiring help from a caregiver and the proportion relying on informal care. The impact of MG-ADL scores on the iCG work status and productivity was based on the same study.^15,30^ The data inputs regarding caregivers applied in the model are summarized in **Table 2**.

**Table 2.** Informal Caregivers of People Living with gMG: Impact on Labor Market by MG-ADL Score

**MG-ADL Score**	**People Living with gMG**	**iCG**	
	**Proportion Needing Help from iCG^a^, %**	**Proportion Stopping Work^b^, %**	**Proportion Reducing Working Hours^c^, %**
<5	10	10	10
5-7	36	15	14
8-9	79	19	18
≥10	100	40	37

Source: Estimates based on MyRealWorld MG Study^20,30^

Abbreviations: gMG, generalized myasthenia gravis; iCG, informal caregivers; MG-ADL, Myasthenia Gravis–Activities of Daily Living.

^a^Based on the odds ratio of patients needing help from a caregiver by MG-ADL score (1.39).

^b^Estimates based on the proportion of iCG (88%), the odds ratio of iCG stopping work or reducing working hours (1.10), and on the proportion stopping work instead of reducing working hours (51%).

^c^Estimates based on the proportion of iCG (88%), the odds ratio of iCG stopping work or reducing working hours (1.10), and on the proportion reducing working hours instead of stopping work (49%).

### Tax Revenue

Tax revenue per cohort was estimated as the expected difference to the average tax revenue collected from those unaffected by the disease – tax revenue loss attributable to gMG. Using a demographically identical cohort from the general population in Canada as the baseline,^25,27^ the model estimates the difference in gMG individuals and iCGs exiting the workforce or experiencing increased levels of absenteeism.

The annual employment income was sourced from sex- and age-specific national statistics^26^ and used to value time on employment. The tax revenue from the working population (patients and iCGs) was estimated by applying the tax wedge rate (31.9%)^31^ to the employment income. The tax wedge includes all tax rates due in the labor market: personal income tax, contributions to the Canada Pension Plan (CPP) and employment insurance (paid by both employers and employees), and provincial-specific payroll taxes. Indirect taxes were also considered and applied to net employment income (proxied as the employment income after tax wedge). The average tax on goods and services, as percentage of gross domestic product (7.3%) was used to estimate the national revenue loss from indirect taxes.^32^

Absenteeism during sick leave represents a loss of corporate revenue and a subsequent loss of tax revenue to both federal and provincial governments. Among iCGs with reduced working hours, the estimated tax revenue loss was based on the average reduction in hours per week reported in the MRW MG study (13 hours/week).^15^ Assuming a marginal productivity of 50%, the tax loss from time on sick leave or caring purposes was based on the national tax revenue as percentage of gross domestic product (33%)^33^ and on the added value per working hour (CAD$73.25).^27^

Income from social benefits were also deducted from indirect taxes, applying the tax on goods and services (as percentage of GDP).^32^

### Benefit Payments

Employed people with gMG taking sick leave (**Table 1**) were assumed to receive sickness benefits for the average time on sick leave.^15,17^ The sick leave period was valued by the maximum amount provided by the Federal Employment Insurance program, per week.^34^

The RR of people living with gMG retiring early or being unable to work because of gMG (**Table 1**) was used to estimate the added proportion of individuals receiving disability benefits or early retirement pensions, by MG-ADL score. The sex- and age-specific rate of people receiving disability benefits in Canada was based on the number of persons with disabilities receiving benefits from the CPP and the overall population below the age of 65 years, in 2012.^35^ After the age of 65, the nonworking cohort was assumed to receive a retirement pension. Nevertheless, as retirement pensions may be requested at age 60,^36^ sex- and age-specific retirement rates and annual pensions applied in the model were sourced from CPP data for those over the age of 60.^26^

Informal caregivers stopping work (**Table 2**)^20,30^ were assumed to receive caregiving benefits, valued by the maximum amount provided by the Federal Employment Insurance program, per week.^37^

A complete list of the fiscal model inputs is presented in **Supplementary Table S2.**

### Sensitivity Analysis

One-way deterministic sensitivity analyses were conducted and summarized in a tornado diagram applying 95% confidence intervals (CIs) to fiscal parameters. In the absence of 95% CIs from the literature, heuristic methods were used to deduct the limits. The gamma distribution was assumed for all cost parameters, marginal productivity, wage growth, and tax rates. Beta distributions were used in rates and proportions. For relative measures, such as RR or odds ratio (OR), the log-normal distribution was assumed. For time-related parameters, the normal distribution was used.

Two scenario analyses were also considered. First, we analyzed how the results would change if treatment had been initiated at the time of diagnosis. Specifically, it was assumed that treatment would be initiated at 37 years of age, according to the age at time of diagnosis in the ADAPT study.^3^ The iCG age was adjusted accordingly to 41 years. A combined conservative scenario was also simulated, considering a reduced impact of disease severity on employment (for both patients and caregivers) and a decreased need for caregiver support. Specifically, it applied a 10% increase in the RR of people living with gMG being employed and the lowest CIs for both the ORs for requiring caregiver assistance and for iCGs stopping work or reducing working hours. Detailed inputs used for this scenario are listed in **Supplementary Table S3**.

## RESULTS

The fiscal consequences of AChR-Ab+ gMG adults whose symptoms persist despite stable doses of SoC medication were estimated from the expected changes in MG-ADL scores, along with the fiscal effects for their greater need for iCGs. The focus of our analysis was on changes in social benefits programs and tax revenue compared with the non gMG general population (**Table 3**). We found that treatment with SoC + efgartigimod resulted in the lowest payment of social benefits compared with the other interventions under study (CAD$79 430). Moreover, lower tax revenue losses were achieved for the cohort receiving SoC + efgartigimod (CAD$117 711) compared with the cohort treated with SoC + chronic IG, the weighted comparator, and SoC alone: CAD$213 370, CAD$219 283 and CAD$237 367, respectively. The greatest overall fiscal burden of disease was observed with SoC + chronic IG with a total lifetime impact of CAD$2 633 339.

**Table 3.** Fiscal Burden of Disease by Treatment Arm over Lifetime Since Treatment Initiation vs Those Unaffected by gMG in the Population

	**Treatment Arm**			
**SoC**	**SoC + Chronic IG**	**Weighted Comparator^a^**	**SoC + EFG**	
Government costs (social transfers), CAD$				
Sickness benefits	17 238	15 872	16 208	15 335
Disability benefits	21 570	20 504	20 767	10 696
Retirement pension	-11 059	-11 275	-11 222	-12 952
Caregiving benefits	144 239	134 986	137 266	66 352
**Total government payments**	**171 988**	**160 087**	**163 019**	**79 430**
Healthcare costs, CAD$				
Drug-related costs	69 214	1 529 289	1 169 560	1 460 633
Disease management costs	759 105	730 592	737 617	464 023
**Total healthcare costs**	**828 319**	**2 259 881**	**1 907 177**	**1 924 656**
Tax revenue loss, CAD$				
Exiting the workforce				
gMG	50 743	48 487	49 042	27 371
iCG	73 358	59 214	62 698	32 793
Absenteeism				
gMG	18 503	16 724	17 162	12 762
iCG	94 764	88 946	90 380	44 786
**Total tax revenue loss**	**237 367**	**213 370**	**219 283**	**117 711**
**Total fiscal consequences to Canadian governments, CAD$**	**1 237 674**	**2 633 339**	**2 289 479**	**2 121 797**

Abbreviations: EFG, efgartigimod; gMG, generalized myasthenia gravis; iCG, informal caregiver; IG, immunoglobulins; SoC, standard of care.

^a^Comprises 75% of patients on chronic IG and 25% on SoC.

Savings in benefit payments and tax revenue losses stemmed from the increase in productive life years (PLYs) of patients and iCGs. Patients receiving efgartigimod were estimated to achieve 13.5 PLYs vs 12.4 PLYs from the cohort on the weighted comparator. A gain of 1.1 PLYs was then expected from efgartigimod relative to the weighted comparator.

**Table 4** shows the lifetime comparative analysis of different therapeutic options that estimates the impact of treatments on different government public accounts. We estimated that the greatest incremental savings in public benefits was observed between SoC alone vs SoC + efgartigimod (CAD$92 556). The smallest incremental gain in benefits payments was observed between SoC + chronic IG vs SoC alone, with CAD$11 900 in public benefits savings. Estimating the comparative lifetime gains in taxes showed the greatest benefit when comparing SoC + efgartigimod with SoC alone, with an incremental gain of CAD$119 656. Similar gains were observed when comparing SoC + efgartigimod with SoC + chronic IG and the weighted comparator (CAD$95 659 and CAD$101 572, respectively by switching to efgartigimod). The comparative lifetime analysis of different therapeutic options showed positive return on investment in efgartigimod compared with chronic IG or the weighted comparator. Specifically, benefit-cost ratios show that efgartigimod is a dominant option compared with chronic IG, resulting in gains of CAD$6.45 for every CAD$1 invested. When efgartigimod was compared with the weighted comparator, a benefit-cost ratio of 1.58 was estimated, suggesting that every CAD$1 invested in efgartigimod may result in savings for the Canadian government of CAD$1.58.

**Table 4.** Lifetime Comparative Fiscal Analysis of Different Therapeutic Options in Canada

	**Treatment Arm**			
**SoC + Chronic IG vs SoC**	**SoC + EFG vs SoC**	**SoC + EFG vs SoC + Chronic IG**	**SoC + EFG vs Weighted Comparator^a^**	
Savings in government payments, CAD$				
Sickness benefits	1365	1902	537	873
Disability benefits	1066	10 874	9808	10 071
Retirement pension	216	1893	1677	1730
Caregiving benefits	9253	77 887	68 634	70 914
**Total savings in government payments^a^ (1)**	**11 900**	**92 556**	**80 656**	**83 588**
Incremental healthcare costs, CAD$				
Drug-related costs (2)	-1 460 075	-1 391 419	68 656	-291 073
Disease management costs (3)	28 513	295 082	266 569	273 594
**Total healthcare costs**	**-1 431 562**	**-1 096 337**	**335 225**	**-17 479**
Savings in tax revenue (ie, averted tax revenue loss), CAD$				
Exiting the workforce				
gMG	2256	23 372	21 116	21 672
iCG	14 144	40 565	26 421	29 906
Absenteeism				
gMG	1779	5741	3962	4400
iCG	5818	49 979	44 160	45 594
**Total savings in tax revenue (4)**	**23 997**	**119 656**	**95 659**	**101 572**
**Total benefits to Canadian governments (1 + 3 + 4), CAD$**	**64 411**	**507 295**	**442 884**	**458 754**
**Benefit-cost ratio (1 + 3 + 4)/(2)**	**0.04**	**0.36**	**6.45^b^**	**1.58**

Abbreviations: gMG, generalized myasthenia gravis; iCG, informal caregiver; SoC, standard of care; IG, immunoglobulins; EFG, efgartigimod.

Negative values represent a cost to Canadian governments. Positive values represent savings to Canadian governments.

^a^Comprises 75% of patients on chronic IG and 25% on SoC.

^b^EFG dominates chronic IG.

### Sensitivity Analysis

**Figure 2.**
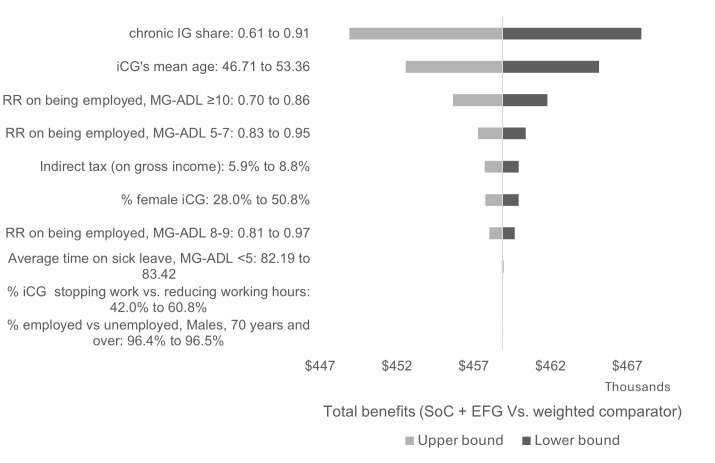
Sensitivity Analysis on Total Benefits to the Canadian Government of SoC + Efgartigimod vs Weighted Comparator Abbreviations: EFG, efgartigimod; iCG, informal caregiver; IG, immunoglobulin; MG-ADL, Myasthenia Gravis – Activities of Daily Living; RR, relative risk; SoC, standard of care.

The 10 most impactful parameters are summarized in a tornado diagram (**Figure 2**), based on the comparison of efgartigimod with the weighted comparator. The parameters with the highest impact on the results are the share of chronic IG in the weighted comparator, iCG age and gender, and RR of being employed according to the MG-ADL score. Nevertheless, the sensitivity analysis suggests that total benefits of using efgartigimod remained substantial across plausible parameter ranges (CAD$≥448 000 per symptomatic adult with AChR-Ab+ gMG).

The scenario analysis assuming efgartigimod initiation at diagnosis estimated total savings of CAD$619 945 per gMG patient and iCG, compared with the weighted comparator. The conservative estimate of reduced impact of disease severity on employment and decreased need for caregiver support led to 2.2% lower total savings per gMG patient and their iCG compared with the weighted comparator, while still yielding a 1.5-fold return on investment in efgartigimod.

For ease of presentation, sensitivity analysis for the other comparisons in the model are not presented in the main manuscript. However, the fiscal parameters presented in **Figure 2** are the most impactful for all comparisons and the reliability of results is preserved.

## DISCUSSION

We applied a fiscal framework by linking MG-ADL scores to the economic burden of gMG to estimate its lifetime impact by treatment option from a governmental perspective. Fiscal consequences were compared in scenarios in which symptomatic AChR-Ab+ patients living with gMG on SoC medication (acetylcholinesterase inhibitors, corticosteroids, and nonsteroidal immunosuppressive therapies) would receive add-on therapies such as chronic IG and efgartigimod. Estimates suggested that investing in an active treatment provide savings to the Canada’s public accounts in disease management costs, benefit payments, and tax revenue losses attributable to gMG. Patients not adding an active treatment (ie, on SoC alone) and their iCGs represent a lifetime cost of CAD$1.24 million to the public sector (including drug-related costs). However, when adding an active treatment, decision-makers must consider lifetime drug-related costs. Both chronic IG and efgartigimod represent an added cost to the public sector when administered to patients receiving SoC alone. In the scenario considering the bundle of treatments administered to symptomatic AChR-Ab+ patients before efgartigimod was available (weighted comparator=25% of patients on SoC alone and 75% additionally on chronic IG),^18^ lifetime savings associated with efgartigimod introduction outweigh incremental drug-related costs. The benefit-cost ratio of reimbursing efgartigimod (vs weighted comparator) was estimated at 1.58 to Canada’s public accounts, suggesting a greater return from each CAD$1 invested in efgartigimod. Estimates are sensitive to the share of people living with gMG receiving chronic IG, as we could expect from the base case analysis, in which efgartigimod was observed as the dominant strategy compared with chronic IG, due to the predicted savings in all fiscal consequences, including drug-related costs, resulting in a benefit-cost ratio of 6.45.

Efgartigimod savings based on improvements in the MG-ADL scale^3^ agree with results in health-related quality-of-life measures and patients achieving minimal symptom expression.^11,14^ This study estimates that, besides diminishing disease management costs, social benefit payments are avoided and tax revenue losses are averted with the introduction of efgartigimod. It is interesting to note that reducing the need for home care of people living with gMG that is inadequately controlled has a major impact on fiscal consequences. The averted tax revenue losses from iCGs preserving their work status and productivity levels^15,17^ and savings in caregiving benefits were estimated to be higher than savings in tax revenue losses and benefit payments due to individuals with gMG. A large proportion of patients rely on working-age family members for caregiving support,^15,20^ and these are commonly of prime working-age (ie, 45-54 years), which creates added fiscal drag.^26^ We applied sensitivity analysis to explore the robustness of how key inputs would influence the modeled conclusions, namely to the share of chronic IG in the weighted comparator, to iCGs demographics and MG-ADL employment risks.

The current analysis shows the broader impact of treating people whose gMG is inadequately controlled by standard therapy. Besides improving quality of life and the financial status of those living with gMG, treatment effectiveness has a major positive impact on household economy and governmental public accounts. From a policy perspective, decision-makers should adopt a holistic approach when evaluating new technologies. Although direct public healthcare costs are often the key criteria for decision making, it is also important to understand and evaluate long-term economic consequences, particularly when treatment effectiveness has a significant impact on the working-age population and government public accounts.

Current estimates are model-based projections that should be interpreted cautiously on their assumptions. The RR of people living with gMG being employed by MG-ADL score and other parameters in the current model, relied solely on the MRW MG study. This was a multicountry prospective, observational, digital, and longitudinal study covering 1693 adults diagnosed with MG.^28^ Nevertheless, the low number of participants by MG-ADL score added uncertainty to the inputs applied in the fiscal model. The current analysis was based on the overall MRW MG study’s sample to reduce uncertainty levels, even though a cohort of Canadian patients could have been identified (n=19). Uncertainty in the RR of gMG individuals being employed by MG-ADL scores, and on the age and sex of iCGs, was responsible for the highest changes in fiscal outcomes. Nevertheless, results were found robust, as varying these inputs would not change the main conclusions.

It is anticipated that those diagnosed with gMG are eligible for novel treatments earlier than the starting age of participants in the ADAPT trial on which the analysis was based.^3^ Therefore, conducting a scenario analysis on the age of treatment start (affecting both patient and iCG) was deemed important. Starting efgartigimod treatment immediately after diagnosis was estimated to increase savings to the public sector by 35% when replacing the weighted comparator. This scenario analysis should nonetheless be considered carefully, as younger patients might be distributed differently by MG-ADL scores and the treatment effect on such a cohort has yet to be assessed.

Other limitations are worth considering when evaluating our findings. First, the inclusion of benefits payments was focused on federal programs. This suggests some additional costs could be applicable at the provincial or municipality level, which would suggest that we underestimated fiscal offsets. Additional limitations to the current work are mainly related to disease modeling and how treatment comparative effectiveness was estimated. The current fiscal model relied on a previously presented cost-effectiveness study whose limitations have been discussed.^10^ It is important to mention that treatment effectiveness of efgartigimod vs chronic IG was based on indirect evidence of changes from baseline in MG-ADL scores, which was not the primary endpoint in the studies considered in the network meta-analysis. Uncertainty in this parameter led to wide 95% CIs regarding comparative effectiveness, suggesting marginal effects. Consistency between trials with respect to dosing regimen, variability in eligibility criteria, and study follow-up times were also previously discussed.^10^

## CONCLUSIONS

Efgartigimod’s introduction in the Canadian market is estimated to save public accounts more than CAD$448 000 per adult with AChR-Ab+ gMG that is not controlled by SoC therapy, compared with a weighted comparator (75% of patients administered SoC medication + chronic IG). Besides savings in disease management costs, avoiding the need for iCGs and enabling relatives to pursue full-time employment prevents the loss of tax revenue while also reducing benefit payments. Clinical improvements allow both people living with gMG and their iCGs to be more productive in the labor market. Evidence of the impact of healthcare technologies on the broader public economy should be made available and considered by decision-makers for evaluating new medical therapies.

## Supplementary Material

Online Supplementary Material
