# Supplementary material for: The Fiscal Consequences for the Canadian Government of Efgartigimod in the Treatment of Generalized Myasthenia Gravis
Source: J Health Econ Outcomes Res. 2026 Mar 11;13(1):85–92. doi: 10.36469/001c.157709 (PMC12986971; doi:10.36469/001c.157709)
Supplement: Online Supplementary Material [file jheor_2026_13_1_157709_334836.pdf]

Journal of Health Economics and Outcomes Research

Online Supplementary Material

The Fiscal Consequences for the Canadian Government of Efgartigimod in the Treatment of Generalized Myasthenia Gravis. *JHEOR*. 2026;13(1):85-92. doi:10.36469/jheor.2026.157709

Figure S1: Health State Transition Diagram

Table S1: Link Between Health States and Labor and Fiscal Stages

Figure S2: Fiscal Model Inputs

Figure S3: Fiscal Model Inputs in Scenario Analysis: Reduced Impact of Disease Severity on Employment, and Decreased Need for Caregiver Support

This supplementary material has been provided by the authors to give readers additional information about their work.

This is an open-access article distributed under the terms of the Creative Commons Attribution 4.0 International License (CCBY-4.0). View this license's legal deed at http://creativecommons.org/licenses/by/4.0 and legal code at http://creativecommons.org/licenses/by/4.0/legalcode for more information.

Figure S1. Health State Transition Diagram

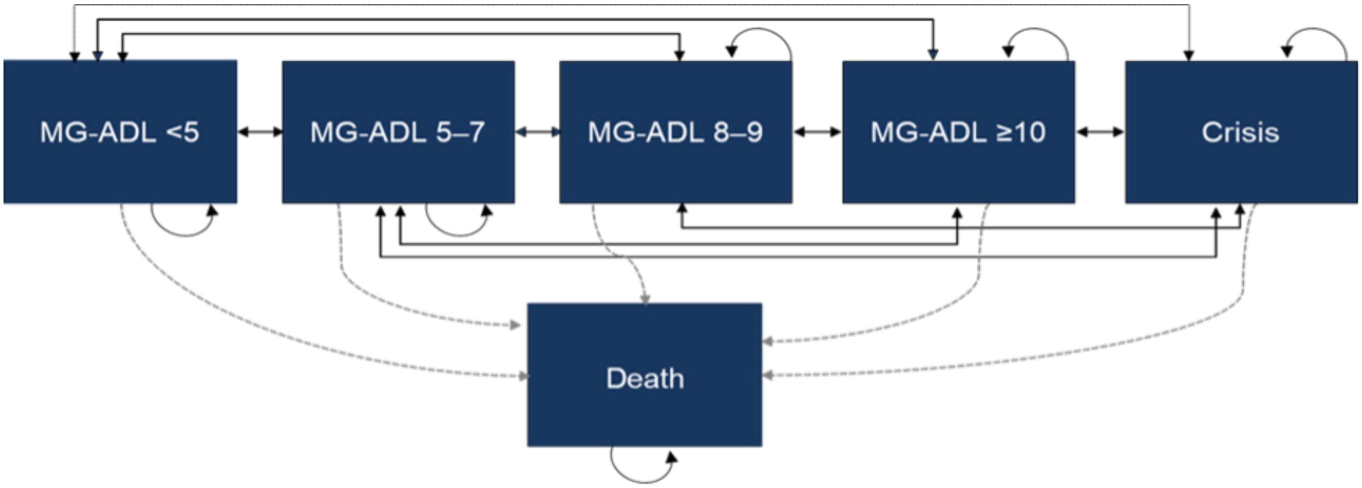

Abbreviation: MG-ADL, Myasthenia Gravis–Activities of Daily Living.

Table S1. Link Between Health States and Labor and Fiscal Stages

**Health States**	**Labor and Fiscal Stages**					
	**Employed**		**Unable to Work Due to Illness**		**Informal Caregiver**	
	**Working**	**Sick Leave**	**Disabled**	**Early Retired**	**Stop Working and Receiving iCG Benefits**	**Working Reduced Hours**
MG-ADL <5<br>MG-ADL 6-7<br>MG-ADL 8-9<br>MG-ADL ≥10	RR on employment by MG-ADL group (ref <5)[1] to age-specific employment rates of GP	OR of patients taking sick leave by MG-ADL score used to estimate % taking sick leave by MG-ADL group	RR on retiring early or unable to work because of gMG by MG-ADL group (ref <5)[1] applied to age-specific % receiving disability or retirement pensions in the GP, respectively.		ORs of patients requiring iCG help by MG-ADL score from MRW-MG,[2] used to estimate the % needing help from a CG by MG-ADL group; % iCG,[3] % iCG stopping to work vs reducing working hours by MG-ADL score from MRW-MG[5]	
gMG crisis	-	Sick leave for 4 weeks applied to all employed in previous cycle	Assumed same proportion of patients receiving disability and retirement pensions in cycle before crisis event.		Assumed same proportion of iCG receiving benefits in cycle before crisis event.	Assumed same reduction of working hours as in MG-ADL ≥10
Death	Death produces loss of direct and indirect tax revenue proportional to life expectancy decreases. Savings from fewer transfers are also quantified.					

Abbreviations: ADL, activities of daily living; CG, caregiver; gMG, generalized myasthenia gravis; GP, general population; iCG, informal caregiver; MRW-MG, MyReal World MG study; OR, odds ratio; ref, reference; RR, relative risk.

Table S2. Fiscal Model Inputs

Parameters	Value	SE	Source
**Fiscal inputs**			
Healthcare costs discount rate	1.5%	NA	Canada's Drug Agency, 2024[6]
Fiscal outcomes discount rate	3.0%	0.003	Government of Canada, 2024[7]
Inflation, CPI	2.5%	0.006	Statistics Canada. Table 18-10-0005-01 Consumer Price Index, annual average, not seasonally adjusted[8]
Wage growth	4.5%	0.005	Statistics Canada. Table 14-10-0064-01 Employee wages by industry, annual[9]
Value added per workhour (CAD$)	73.25	7.325	Statistics Canada. Table 36-10-0480-01 Labour productivity and related measures by business sector industry and by non-commercial activity consistent with the industry accounts[10]
Working hours per day	6.77	0.677	
Labor marginal productivity	50%	0.050	Assumption
Tax wedge	31.9%	0.032	OECD, Taxing wages - Canada 2023[11]
Indirect tax rate (on gross income)	7.3%	0.007	OECD (2024), Tax on goods and services (indicator). doi: 10.1787/40b85101-en (Accessed April 4, 2024)[12]
Tax-GDP ratio	33.0%	0.033	OECD (2024), Tax revenue (indicator). doi: 10.1787/d98b8cf5-en (Accessed April 12, 2024)[13]
gMG age, y	46.9	1.350	ADAPT [14]
Informal CG mean age, y	50	1.700	Estimated from the age difference between patients and CGs in Dewilde 2025[15]
Oldest age on employment income, y	75	NA	Assumption
Oldest age on disability benefits, y	65	NA	Gov Canada 2024: CPP Disability Pension[16]
Youngest age on retirement, y	60	NA	Gov Canada 2024: CPP Retirement Pension[17]
Regular retirement age, y	65	NA	Assumption
RR of being employed, by MG-ADL score			
MG-ADL <5	1.00	Reference	MyRealWorld MG[5]
MG-ADL 5-7	0.89	0.036	
MG-ADL 8-9	0.89	0.045	
MG-ADL ≥10	0.78	0.053	
RR of retiring early or unable to work because of gMG, by MG-ADL score			
MG-ADL <5	1.00	Reference	MyRealWorld MG[5]
MG-ADL 5-7	3.21	0.162	
MG-ADL 8-9	3.53	0.199	
MG-ADL ≥10	5.00	0.147	
OR of taking sick leave by continuous MG-ADL score	1.09	0.019	Dewilde 2025[2]
Proportion taking sick leave, by MG-ADL score			
MG-ADL <5	19%	NA	MyRealWorld MG[5]
MG-ADL 5-7	27%	NA	
MG-ADL 8-9	33%	NA	
MG-ADL ≥10	63%	NA	
Average time on sick leave, by MG-ADL score (hours/4-week cycle)			
MG-ADL <5	82.81	0.315	Based on average number of days of sick leave per month, MyRealWorld MG[5]
MG-ADL 5-7	83.78	0.176	
MG-ADL 8-9	83.78	0.176	
MG-ADL ≥10	133.82	0.559	
OR per 1-point increase in MG-ADL score for requiring CG assistance	1.39	0.030	Dewilde 2025[2]
Proportion of patients requiring help from a CG, by MG-ADL score			

Table S2. Fiscal Model Inputs

Parameters	Value	SE	Source
MG-ADL <5	10%	NA	MyRealWorld MG[5]
MG-ADL 5-7	36%	NA	
MG-ADL 8-9	79%	NA	
MG-ADL ≥10	100%	NA	
Proportion of informal CGs	88%	0.010	Dewilde 2023[3]
OR per 1-point increase in MG-ADL score for informal CGs stopping work or reducing working hours	1.10	0.04	Dewilde 2025[2]
Proportion of informal CGs stopping work vs reducing working hours	0.51	0.05	MyRealWorld MG[5]
Proportion of informal CGs stopping work, by MG-ADL score			
MG-ADL <5	10%	NA	MyRealWorld MG[5]
MG-ADL 5-7	15%	NA	
MG-ADL 8-9	19%	NA	
MG-ADL ≥10	40%	NA	
Proportion of informal CGs reducing working hours, by MG-ADL score			
MG-ADL <5	10%	NA	MyRealWorld MG[5]
MG-ADL 5-7	14%	NA	
MG-ADL 8-9	18%	NA	
MG-ADL ≥10	37%	NA	
Proportion of female informal CGs	39%	0.06	Dewilde 2025[15]
Average working hours reduced by the informal CG (per 4-week cycle)	52.00	0.66	Dewilde 2023[3]
**Labor market inputs**			
Annual employment income, by sex and age group (CAD$)			
Males			Statistics Canada. Table 11-10-0239-01 Income of individuals by age group, sex and income source, Canada, provinces and selected census metropolitan areas[18]
35-44 years	77 900	7790	
45-54 years	82 700	8270	
55-64 years	67 600	6760	
≥65 years	27 600	2760	
Females			
35-44 years	52 800	5280	
45-54 years	61 300	6130	
55-64 years	45 600	4560	
≥65 years	21 700	2170	
Participation rate, by sex and age group			
Males			Statistics Canada. Table 14-10-0327-01 Labour force characteristics by sex and detailed age group, annual[19]
35-39 years	93%	0.00	
40-44 years	93%	0.00	
45-49 years	93%	0.00	
50-54 years	91%	0.00	
55-59 years	82%	0.00	
60-64 years	65%	0.00	
65-69 years	36%	0.00	
≥70 years	11%	0.00	

Table S2. Fiscal Model Inputs

**Parameters**	**Value**	**SE**	**Source**
Females			Statistics Canada. Table 14-10-0327-01 Labour force characteristics by sex and detailed age group, annual[19]
35-39 years	85%	0.00	
40-44 years	87%	0.00	
45-49 years	85%	0.00	
50-54 years	84%	0.00	
55-59 years	74%	0.00	
60-64 years	53%	0.00	
65-69 years	23%	0.00	
≥70 years	6%	0.00	
Proportion of employed vs unemployed, by sex and age group			
Males			Statistics Canada. Table 14-10-0327-01 Labour force characteristics by sex and detailed age group, annual[19]
35-39 years	96%	0.00	
40-44 years	96%	0.00	
45-49 years	97%	0.00	
50-54 years	96%	0.00	
55-59 years	96%	0.00	
60-64 years	94%	0.00	
65-69 years	95%	0.00	
≥70 years	96%	0.00	
Females			
35-39 years	96%	0.00	
40-44 years	96%	0.00	
45-49 years	96%	0.00	
50-54 years	96%	0.00	
55-59 years	96%	0.00	
60-64 years	95%	0.00	
65-69 years	96%	0.00	
≥70 years	97%	0.00	
Proportion of employed in public sector, by sex			
Males	15%	NA	Statistics Canada. Table 14-10-0027-01 Employment by class of worker, annual (×1000)[20]
Females	28%	NA	
Benefit payments inputs			
Proportion of employed receiving sickness benefits, by sex and age group			
Males			Statistics Canada. Table 14-10-0009-01 Employment insurance beneficiaries by type of income benefits, monthly, unadjusted for seasonality[21]
30-54 years	4%	NA	
55-64 years	7%	NA	
Females			
30-54 years	7%	NA	
55-64 years	10%	NA	
Proportion receiving disability benefits, by sex and age group			
Males			Statistics Canada. Table 13-10-0358-01 Canada and Quebec pension plan benefits of adults with and without disabilities[22]
35-44 years	0%	NA	
45-54 years	2%	NA	
55-64 years	8%	NA	
≥65 years	29%	NA	

Table S2. Fiscal Model Inputs

**Parameters**	**Value**	**SE**	**Source**
Females			Statistics Canada. Table 13-10-0358-01 Canada and Quebec pension plan benefits of adults with and without disabilities[22]
35-44 years	1%	NA	
45-54 years	3%	NA	
55-64 years	8%	NA	
≥65 years	30%	NA	
Proportion receiving retirement pension, by sex and age group (CPP)			
Males			Statistics Canada. Table 11-10-0239-01 Income of individuals by age group, sex and income source, Canada, provinces and selected census metropolitan areas[18]
35-44 years	1%	NA	
45-54 years	2%	NA	
55-64 years	23%	NA	
≥65 years	93%	NA	
Females			
35-44 years	1%	NA	
45-54 years	4%	NA	
55-64 years	27%	NA	
≥65 years	89%	NA	
Sickness benefit, amount per week (CAD$)	668	NA	Government of Canada, Employment Insurance benefits and leave[23]
Disability benefits, average monthly amount (CAD$)			
CPP disability benefit	1177	NA	Government of Canada, Canada Pension Plan disability benefits-Average monthly amount for new beneficiaries (Oct. 2023)[16]
CPP post-retirement disability benefit	583	NA	
Retirement pensions (CPP), by sex and age group, average amount per year (CAD$)			
Males			Statistics Canada. Table 11-10-0239-01 Income of individuals by age group, sex and income source, Canada, provinces and selected census metropolitan areas[18]
60-64 years	8100	NA	
≥65 years	9000	NA	
Females			
60-64 years	6900	NA	
≥65 years	7600	NA	
Caregiving benefits, amount per week (CAD$)	668	NA	Government of Canada, Caregiving benefits[24]
Average working hours lost among the general population, per year, by reason			
Illness or disability			Statistics Canada. Table 14-10-0196-01 Work absence of full-time employees by public and private sector, annual[25]
Males	49.5	NA	
Females	71.7	NA	
Personal or family responsibility			
Males	11.6	NA	
Females	16.5	NA	

Abbreviations: CG, caregiver; CPI, consumer price index; CPP, Canadian Pension Plan; GDP, gross domestic product; gMG, generalized myasthenia gravis; GP, general population; iCG, informal caregiver; MG, myasthenia gravis; MG-ADL, Myasthenia Gravis–Activities of Daily Living; NA, not applicable; OECD, Organisation for Economic Co-operation and Development; OR, odds ratio; RR, relative risk; SE, standard error.

Table S3. Fiscal Model Inputs in Scenario Analysis: Reduced Impact of Disease Severity on Employment and Decreased Need for Caregiver Support

Parameters	Base Case	Difference Applied to Scenario Analysis	Scenario
RR of being employed, by MG-ADL score	–	–	–
MG-ADL <5	1.00	–	1.00
MG-ADL 5-7	0.89	+10%	0.98
MG-ADL 8-9	0.89	+10%	0.98
MG-ADL ≥10	0.78	+10%	0.85
OR per 1-point increase in MG-ADL score for requiring caregiver assistance	1.39	Lowest 95% CI	1.32
OR per 1-point increase in MG-ADL score for informal caregivers stopping work or reducing working hours	1.10	Lowest 95% CI	1.02
Results for efgartigimod vs weighted comparator			
Total benefits to the Canadian government	458 754	–	448 742
Benefit-cost ratio	1.58	–	1.54

Abbreviations: CI, confidence interval; MG-ADL, Myasthenia Gravis–Activities of Daily Living; OR, odds ratio; RR, relative risk.

REFERENCES

1. Berrih-Aknin S, Palace J, Meisel A, et al. Patient-reported impact of myasthenia gravis in the real world: findings from a digital observational survey-based study (MyRealWorld MG). *BMJ Open*. 2023;13(5):e068104. doi:10.1136/bmjopen-2022-068104
2. Dewilde S, Qi ZC, Femke DR, et al. A cost analysis of reductions in work productivity for MG patients and their caregivers by symptom severity. *Front Public Health*. 2025;13:1538789 doi:10.3389/fpubh.2025.1538789
3. Dewilde S, Phillips G, Paci S, De Ruyck F, Tollenaar NH, Janssen MF. People diagnosed with myasthenia gravis have lower health-related quality of life and need more medical and caregiver help in comparison to the general population: analysis of two observational studies. *Adv Ther*. 2023;40(10):4377-4394. doi:10.1007/s12325-023-02604-z
4. Jacob S, Dewilde S, Qi C, et al. Productivity losses for MG patients and their caregivers–association with disease severity. 2024:
5. Data on file. MyRealWorld MG. 2022.
6. Procedures for Reimbursement Reviews (Canada's Drug Agency - L'Agence des Médicaments du Canada, CDA-AMC) 1-196 (2024).
7. Government of Canada. Canada's Cost-Benefit Analysis Guide for Regulatory Proposals. 2024. Accessed April 5, 2024. https://www.canada.ca/en/government/system/laws/developing-improving-federal-regulations/requirements-developing-managing-reviewing-regulations/guidelines-tools/cost-benefit-analysis-guide-regulatory-proposals.html
8. Statistics Canada. Table 18-10-0005-01 Consumer Price Index, annual average, not seasonally adjusted. 2024. Accessed April 4, 2024. https://www150.statcan.gc.ca/t1/tbl1/en/tv.action?pid=1810000501
9. Statistics Canada. Table 14-10-0064-01 Employee Wages by Industry, Annual. 2024. Accessed April 4, 2024. https://www150.statcan.gc.ca/t1/tbl1/en/tv.action?pid=1410006401
10. Statistics Canada. Table 36-10-0480-01 Labour productivity and related measures by business sector industry and by non-commercial activity consistent with the industry accounts. 2024. Accessed April 12, 2024. https://www150.statcan.gc.ca/t1/tbl1/en/tv.action?pid=3610048001
11. OECD. Taxing Wages–Canada 2023. https://www.oecd.org/tax/tax-policy/taxing-wages-canada.pdf
12. OECD. Tax on Goods and Services, as a Percentage of Gross Domestic Product. 2024. Accessed April 4, 2024. https://data.oecd.org/tax/tax-on-goods-and-services.htm#indicator-chart
13. OECD. Tax Revenue (Indicator). 2024. Accessed April 12, 2024. https://data.oecd.org/tax/tax-on-goods-and-services.htm#indicator-chart
14. Howard JF Jr, Bril V, Vu T, et al. Safety, efficacy, and tolerability of efgartigimod in patients with generalised myasthenia gravis (ADAPT): a multicentre, randomised, placebo-controlled, phase 3 trial. *Lancet Neurol.* 2021;20(7):526-536. doi:10.1016/S1474-4422(21)00159-9
15. Dewilde S, Tollenaar N, Boulanger P, et al. Caregiving burden among caregivers of people with myasthenia gravis. *Orphanet J Rare Dis.* 2025;20(1):311.
16. Government of Canada. Canada Pension Plan disability benefits: long-term or permanent disability. Accessed April 3, 2024. https://www.canada.ca/en/services/benefits/publicpensions/cpp/cpp-disability-benefit/benefit-amount.html
17. Government of Canada. Old age security pension. Accessed April 3, 2024. https://www.canada.ca/en/services/benefits/publicpensions/cpp/old-age-security.html
18. Statistics Canada. Table 11-10-0239-01 Income of individuals by age group, sex and income source, Canada, provinces and selected census metropolitan areas. 2023. Accessed April 4, 2024. https://www150.statcan.gc.ca/t1/tbl1/en/tv.action?pid=1110023901
19. Statistics Canada. Table 14-10-0327-01 Labour force characteristics by sex and detailed age group, annual. 2024. annual. Accessed April 1, 2024. https://www150.statcan.gc.ca/t1/tbl1/en/tv.action?pid=1410032701
20. Statistics Canada. Table 14-10-0027-05 Employment by class of worker, annual (x1,000). 2024.
21. Statistics Canada. Table 14-10-0009-01 Employment insurance beneficiaries by type of income benefits, monthly, unadjusted for seasonality. 2024. Accessed April 3, 2024. https://www150.statcan.gc.ca/t1/tbl1/en/tv.action?pid=1410000901
22. Statistics Canada. Table 13-10-0358-01 Canada and Quebec pension plan benefits of adults with and without disabilities. 2015. Accessed April 3, 2024. https://www150.statcan.gc.ca/t1/tbl1/en/tv.action?pid=1310035801
23. Government of Canada. Employment Insurance benefits and leave. Accessed April 3, 2024. https://www.canada.ca/en/services/benefits/ei/ei-sickness.html
24. Government of Canada. Caregiving benefits. Accessed April 3, 2024. https://www.canada.ca/en/services/benefits/ei/caregiving.html
25. Statistics Canada. Table: 14-10-0196-01 Work absence of full-time employees by public and private sector, annual. 2024.
